# Folate intake, *MTHFR* genotype and premenstrual symptoms

**DOI:** 10.1017/S0007114525103620

**Published:** 2026-03-28

**Authors:** Tara Zeitoun, Ahmed El-Sohemy

**Affiliations:** Department of Nutritional Sciences, Temerty Faculty of Medicine, University of Toronto, Toronto, ON M5S 3H2, Canada

**Keywords:** Premenstrual symptoms, Folate, *MTHFR* genotype, Nutrigenomics, Premenstrual depression, Women’s health

## Abstract

Premenstrual symptoms are a cyclic set of symptoms that affect women’s psychological and physical well-being. Growing evidence suggests that micronutrients may contribute to the risk and severity of premenstrual symptoms such as depression. Yet the relationship between folate and premenstrual symptoms remains inconclusive. The objective of this study was to determine the association between folate intake and *MTHFR* genotype with premenstrual symptoms. Females (*n* 678) aged 20–29 years from the Toronto Nutrigenomics and Health Study self-reported fifteen premenstrual symptoms. Dietary intake was measured using a validated 196-item Toronto-modified Harvard food frequency questionnaire. DNA was isolated from peripheral white blood cells and genotyped for the C677T *MTHFR* (rs1801133) polymorphism. Using logistic regression, the odds of experiencing premenstrual symptoms were compared between total folate intake below and above the median (647 mcg/d) and between *MTHFR* genotypes. We found associations between *MTHFR* genotype and some premenstrual symptoms. Among women with low folate intake, an additive association was observed between the Tallele of *MTHFR* and premenstrual depression. Compared with those with the CC genotype, the OR (95 % CI) for depression was 1·66 (0·98, 2·87) for those with the CT genotype and 2·41 (1·08, 5·38) for those with the TT genotype. No associations were observed between *MTHFR* genotype and premenstrual depression among those with higher habitual intakes of folate. Since the *MTHFR* genotype is involved in the folate metabolic pathway, these findings suggest that folate or its metabolites may be related to the risk of premenstrual depression.

Premenstrual symptoms are a collection of adverse psychological and somatic symptoms that are experienced by most females of reproductive age^(1)^. These symptoms occur during the luteal phase of the menstrual cycle, usually 5–12 d before menstruation, and subside after the onset of menses. These reoccurring symptoms can adversely impact personal, social and vocational domains^(2)^. Premenstrual symptoms occur within two premenstrual disorders: premenstrual syndrome (PMS) and premenstrual dysphoric disorder (PMDD). PMS is defined as having at least one moderate/severe psychological and one somatic symptom and affects approximately 30 % of women^(3)^, whereas PMDD is characterised by having at least five symptoms, one of which must be psychological, and is seen in up to 8 % of women^(4)^.

Previous randomised controlled clinical trials have tested the efficacy of vitamin B_6_ in alleviating PMS when compared with placebo in which a few studies have found that up to 100 mg of vitamin B_6_ daily may reduce the occurrence and severity of some premenstrual symptoms^(5–7)^. Research on other B vitamins, specifically folate, has not been as extensively investigated and the few studies that have assessed this association have been inconsistent^(8,9)^. Such inconsistencies may be due to the shortcomings of relying on dietary assessment methods for micronutrients that have a more complex absorption, such as folate. An individual’s ability to metabolise dietary folate efficiently depends, in part, on the *MTHFR* gene^(10)^. The *MTHFR* gene codes for methylenetetrahydrofolatereductase (MTHFR), which is a key enzyme in folate metabolism. MTHFR converts 5,10-methylenetetrahydrofolate from the diet to 5-methyl-tetrahydrofolate, which is the active form of folate used at the cellular level. Several studies have demonstrated that folate status indicators are influenced by common genetic polymorphisms and that such polymorphisms are partly responsible for the variation in biomarker concentrations in individuals^(11–13)^.

Folate is an essential water-soluble B vitamin that must be obtained from the diet. As of November 1998, folic acid fortification on all flour products, enriched pasta and cornmeal became mandatory in Canada for neural tube defect prevention^(14)^. Folate provides one carbon derivatives that are used for nucleotide synthesis and methylation reactions^(15,16)^. This vitamin is also associated with the formation of the cofactor S-adenosyl-methionine and tetrahydrobiopterin, which are required for serotonin and dopamine metabolism^(17)^. Since folate is used as a co-factor for neurotransmitters, folate deficiency is often manifested in the form of psychiatric condition symptomology such as depression. The effect of folate on depression has been thoroughly investigated, with meta-analyses examining this association suggesting that folate supplementation may be beneficial for individuals with depression^(18–20)^. The relationship between folate and depression may give us insight into folate’s role in potentially reducing premenstrual symptoms, specifically psychological symptoms. The objective of this study was to investigate the association between folate, *MTHFR* genotype and premenstrual symptoms.

## Methods

### Study population

Participants were from the Toronto Nutrigenomics and Health (TNH) study, which is a cross-sectional study examining the effects of genetic variation and dietary intake on biomarkers of health. The TNH study consists of a multiethnic population of young adults and recruitment occurred between 2004 and 2010 at the University of Toronto campus. Participants who met the inclusion criteria provided written informed consent, and the study protocol was approved by the University of Toronto’s Research Ethics Board. Women who were pregnant or breastfeeding were not eligible to participate.

**Figure 1.**
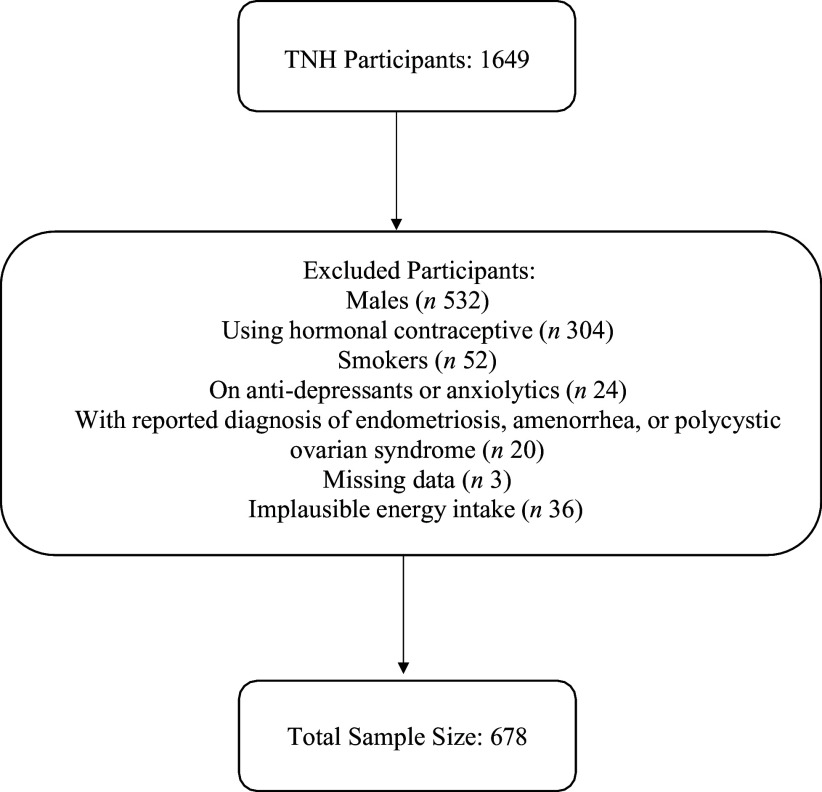
Figure 1 long description. Participant flowchart summarising exclusion criteria for the present study. TNH, Toronto Nutrigenomics and Health Study.

For the present analyses, male participants (*n* 532) were excluded (Figure 1). Those who reported a diagnosis of endometriosis, amenorrhea or polycystic ovarian syndrome (*n* 20), smokers (*n* 52), users of hormonal contraceptives (*n* 304) and users of anxiolytics or anti-depressants (*n* 24) were also excluded due to the potential confounding effects of these variables on premenstrual symptoms or folate status. Those with missing data from the General Health and Lifestyle Questionnaire (GHLQ), or missing genetic data were also excluded (*n* 3). Individuals who may have underreported (< 2092 kJ/day (500 kcal/day) kcal/d) or overreported (> 14644 kJ/day (3500 kcal/d)) their energy intakes were excluded (*n* 36). Based on their self-reported ethnocultural status, the remaining 678 participants were divided into four major ethnic groups: Caucasian (*n* 243), East Asian (*n* 212), South Asian (*n* 78) and Other (*n* 45). Caucasians were those who identified as European, Middle Eastern, or Hispanic. East Asians included people who self-reported from China, Korea, Japan, Philippines, Vietnam, Thailand and Cambodia. South Asians consisted of Bangladeshis, Sri Lankans, Indians and Pakistanis. Aboriginal Canadians, Afro-Caribbeans and those who self-reported belonging to two or more ethnic groups were included in the Other category.

### General health and lifestyle questionnaire

A GHLQ, which consisted of a comprehensive set of questions examining age, sex, ethnocultural group, current medical conditions, medication use, hormonal contraceptive use, dietary supplements, special diets and physical activity levels, was completed by all participants^(21,22)^. The GHLQ contained a premenstrual symptoms questionnaire, as previously described^(23)^, that assessed the severity and presence of fifteen commonly reported premenstrual symptoms within 5 d of the onset of menstruation and up to 4 d afterwards. There were four severity levels: ‘none’, ‘mild’, ‘moderate’ and ‘severe’ that were self-reported by all female participants. This questionnaire was developed by combining the most frequently reported symptoms in the literature and previously validated questionnaires^(24,25)^. The questionnaire was used in several previous studies^(1,21–23,26,27)^.

Recent research on mental health conditions supports a shift from broad syndromic classifications to more specific symptoms and subtypes^(28)^, reinforcing our methodological choice of assessing symptoms individually. Given the heterogeneity of PMS classification, analysing individual symptoms allows for a more precise understanding of their distinct biological, hormonal and genetic mechanisms. As each woman experiences a unique combination of symptoms, this approach better captures the complexity of premenstrual symptoms rather than assuming a uniform aetiology for multiple symptoms as a single condition.

Trained personnel took anthropometric measurements such as weight, height and waist circumference while participants wore light clothing and had their shoes removed. BMI was calculated using these measurements (weight(kg)/height(m)^2^). The GHLQ also included self-reported physical activity, with participants estimating their own activity levels, which included time spent sleeping and engaging in light to vigorous activity. These values were then converted to weekly metabolic hour equivalents (met-h/wk).

### Dietary assessment and genotyping

A validated 196-item Toronto-modified Harvard FFQ was used to assess habitual intake over the previous month, including foods, beverages and supplements^(29)^. As previously described, participants’ responses to each individual item were used to calculate nutrient intakes using the nutrient contents of the food in the US Department of Agriculture’s database^(30)^. For the analyses presented here, folate intakes from food and supplements were calculated using data from the FFQ. By selecting from several frequency options, subjects estimated their consumption of a predetermined portion of each item over the previous month. Following that, responses were converted into estimated daily averages of total folate intake from foods and supplements.

### Genotyping

All participants provided an overnight 12-hour fasting blood sample, and DNA was extracted from whole blood cells using previously described methods at LifeLabs medical laboratory services (Toronto, Canada)^(31)^. DNA was extracted from white blood cells in the peripheral circulation. *MTHFR* (rs1801133) genotyping (C677T) was performed on all participants using Sequenom MassARRAY® technology, as previously described^(32)^ at the Clinical Genomics Centre in Princess Margaret Hospital (Toronto, Canada).

### Statistical analyses

R and RStudio were used for all statistical analyses (version 4.2.0). The distribution of continuous variables was evaluated for normality prior to analysis. BMI was not normally distributed and was thus log-transformed for all analysis. The *α* was set at 0·05, and all *P*-values reported are two-sided. In this study, severities were classified as ‘present’ (mild, moderate or severe) or ‘absent’ (none) to examine the prevalence of premenstrual symptoms. Subject characteristics were compared between participants with above and below the median folate intake (647 mcg/d) using analysis of variance for continuous variables and chi-square tests for categorical variables. The recommended daily allowance of folate intake for females over 19 years of age is 400 mcg/d, and the upper limit is 1000 mcg/d^(33)^.

Binomial logistic regressions were used to determine the association between folate intake and *MTHFR* genotypes and premenstrual symptoms, separately. Logistic regressions were also employed to determine whether *MTHFR* genotype modified the association between folate intake and the presence of premenstrual symptoms. OR and corresponding 95 % CI were calculated to compare the odds of experiencing premenstrual symptoms for participants with low *v*. high folate intake (i.e. below and above the median of 647 mcg/d) using ‘no symptoms’ as the reference category, and between *MTHFR* genotypes, using the CC genotype as the reference category. OR and 95 % CI were also calculated to compare the odds of experiencing premenstrual symptoms in low *v*. high folate intake stratified by *MTHFR* genotype. For each symptom, the *P*-values for the interaction term between *MTHFR* and folate intake were calculated. Univariate and multivariate analyses were performed with adjustments for age, ethnicity, log-transformed BMI, physical activity, total vitamin B_12_ intake and energy intake. The interaction term *P*-values were subjected to Benjamini–Yekutieli adjustments for multiple comparisons (15 tests, *α* = 0·05: *P* < 0·015).

Although genetic variants typically have a small effect size on the outcome observed, the *MTHFR* genotype is strongly associated with folate status^(10–12)^. Thus, the statistical analyses performed in this study were adequately powered, even with a small effect size of 0·2^(34)^. Sample size was determined using a power of 0·8, a small effect size of 0·2 and a significance of *P* < 0·05 using R (Version 4.0.3) and RStudio (version 1.3.1). Based on this equation, the observed sample sizes in previous studies assessing *MTHFR* and other outcomes^(13,35,36)^, an adequate sample size is estimated to be approximately 190 participants.

## Results

Participant characteristics were stratified by *MTHFR* genotype and are presented in Table 1. Most participants had the CC genotype (47 %), followed by the CT (42 %) and TT (11 %) genotypes. There were no significant differences between genotypes for age, BMI, physical activity level, total folate intake and total vitamin B_12_ intake. Energy intake was significantly higher among those with the TT genotype, compared with the CT and CC genotypes (*P* = 0·03). Protein and total fat intake were also significantly higher among those with the TT genotype in comparison to the CT and CC genotype carriers (*P* = 0·008 and 0·05, respectively).

**Table 1.** Participant characteristics stratified by *MTHFR* genotype^*^ (Numbers and percentages; mean values and standard deviations) Table 1 long description.

	CC		CT		TT		*P*^†^
	*n*	%	*n*	%	*n*	%	
*n*, (%)	316	47	286	42	76	11	
	Mean	sd	Mean	sd	Mean	sd	
Age, (years)	22·4	2·5	22·4	2·4	22·33	2·59	0·94
	*n*	%	*n*	%	*n*	%	
Ethnicity, *n* (%)							0·001
Caucasian	96	40	115	47	32	13	
East Asian	128	41	148	47	36	11	
South Asian	59	76	15	19	4	5	
Other	33	73	8	18	4	9	45
	Mean	sd	Mean	sd	Mean	sd	
BMI (kg/m^2^)	22·42	3·68	22·14	3·14	21·41	2·82	0·06
Physical activity level (MET-h/week)^‡^	1·74	0·39	1·73	0·44	1·67	0·37	0·38
Energy (kcal/d)	1781	609	1884	622	1953	673	0·03
Carbohydrates (g/d)	240	92	247	90	260	98	0·21
Total fat (g/d)	58	24	64	27	65	27	0·008
Protein (g/d)	78	29	83	33	84	31	0·05
Thiamin (mg/d)	2·3	5·1	2·7	6·0	2·0	1·1	0·58
Riboflavin (mg/d)	2·9	5·2	3·2	6·1	2·5	1·3	0·53
Niacin (mg/d)	26·2	13·6	27·9	18·7	27·4	14	0·42
Pantothenic acid (mg/d)	7·8	6·8	8·6	8·5	7·4	3·7	0·30
Vitamin B_6_ (mg/d)	4·1	13·2	4·7	14·4	4·9	17·8	0·84
Folate (mcg/d)	780	488	776	464	755	420	0·91
Vitamin B_12_ (mg/d)	7·4	7·4	8·4	9·3	6·7	3·9	0·14

* Values are unadjusted means (standard deviations) for continuous variables unless otherwise indicated.

† Differences between groups were compared using chi-square tests for categorical variables and ANOVA for continuous variables.

‡ MET: metabolic equivalent.

Table 2 presents the associations among total folate intake, stratified by the median (647 mcg/d) and individual premenstrual symptoms. There were no observed associations between folate intake and any premenstrual symptoms, in both univariate and multivariate models, adjusting for age, log-transformed BMI, ethnicity, physical activity level, energy intake and total vitamin B_12_ intake.

**Table 2.** Associations between higher folate intake and risk of premenstrual symptoms (Numbers and percentages; OR and 95 % CI) Table 2 long description.

Premenstrual symptom, *n* (%)	Folate intake < 651 mcg/d (*n* 337)	Folate intake ≥ 651 mcg/d (*n* 341)	Unadjusted OR 95 CI	Unadjusted *P*	Adjusted OR 95 CI^*^	Adjusted P^*^
Acne/skin blemish						
No	146 (43 %)	129 (38 %)	REF		REF	
Yes	191 (57 %)	212 (62 %)	0·8 (0·59, 1·08)	0·15	0·83 (0·58, 1·18)	0·3
Desire to be alone						
No	218 (65 %)	216 (63 %)	REF		REF	
Yes	119 (35 %)	125 (37 %)	0·94 (0·69, 1·29)	0·72	0·92 (0·64, 1·32)	0·66
Anxiety/tension/nervousness						
No	210 (62 %)	216 (64 %)	REF		REF	
Yes	127 (38 %)	125 (36 %)	1·05 (0·76, 1·43)	0·78	1·26 (0·88, 1·8)	0·21
Increased appetite/food cravings						
No	130 (39 %)	124 (36 %)	REF		REF	
Yes	207 (61 %)	217 (64 %)	0·91 (0·67, 1·24)	0·55	0·87 (0·61, 1·25)	0·46
Bloating/swelling/breast tenderness						
No	97 (29 %)	89 (26 %)	REF		REF	
Yes	240 (71 %)	252 (74 %)	0·87 (0·62, 1·23)	0·43	0·9 (0·61, 1·33)	0·61
Clumsiness						
No	286 (85 %)	286 (84 %)	REF		REF	
Yes	51 (15 %)	55 (16 %)	0·93 (0·61, 1·4)	0·72	1·19 (0·74, 1·94)	0·47
Confusion/difficulty concentrating/forgetfulness						
No	261 (78 %)	263 (77 %)	REF		REF	
Yes	76 (23 %)	78 (23 %)	0·98 (0·69, 1·41)	0·92	1·12 (0·74, 1·7)	0·6
Cramps						
No	80 (24 %)	76 (22 %)	REF		REF	
Yes	257 (76 %)	265 (8 %)	0·92 (0·64, 1·32)	0·65	0·98 (0·65, 1·49)	0·94
Depression						
No	245 (73 %)	239 (70 %)	REF		REF	
Yes	92 (27 %)	102 (30 %)	0·88 (0·63, 1·23)	0·45	0·98 (0·67, 1·44)	0·92
Fatigue						
No	145 (43 %)	157 (46 %)	REF		REF	
Yes	192 (57 %)	184 (54 %)	1·13 (0·83, 1·53)	0·43	1·02 (0·72, 1·45)	0·91
Headaches						
No	257 (76 %)	243 (71 %)	REF		REF	
Yes	80 (24 %)	98 (29 %)	0·77 (0·55, 1·09)	0·14	0·78 (0·52, 1·16)	0·22
Insomnia						
No	298 (88 %)	303 (89 %)	REF		REF	
Yes	39 (12 %)	38 (11 %)	1·04 (0·65, 1·68)	0·86	1·19 (0·69, 2·06)	0·53
Mood swings/crying easily/irritability/angry outbursts						
No	94 (28 %)	92 (27 %)	REF		REF	
Yes	243 (72 %)	249 (73 %)	0·96 (0·68, 1·34)	0·79	1·08 (0·73, 1·59)	0·7
Nausea						
No	288 (85 %)	291 (85 %)	REF		REF	
Yes	49 (15 %)	50 (15 %)	0·99 (0·65, 1·52)	0·96	1·08 (0·66, 1·78)	0·77
Sexual desire/activity change						
No	188 (56 %)	166 (49 %)	REF		REF	
Yes	149 (44 %)	175 (51 %)	0·75 (0·56, 1·02)	0·06	0·81 (0·57, 1·15)	0·25

* *P* value adjusted for age, log-transformed BMI, ethnicity, physical activity level, energy intake and vitamin B_12_ intake.

Excluded: smokers, those with a hormonal imbalance diagnosis, HC users, those on antidepressants or anxiolytics, those with an implausible energy intake (< 500 or > 3500 kcal/d), and individuals with missing information.

The associations between total folate intake and individual premenstrual symptoms stratified by *MTHFR* genotype are shown in Table 3. There were no associations between folate intake and any premenstrual symptoms when stratified by *MTHFR* genotype. Moreover, *MTHFR* did not modify the association between folate intake and premenstrual symptoms after adjusting for covariates, as shown in Table 4.

**Table 3.** Associations between higher folate (> 647 mcg/d) intake and premenstrual symptoms stratified by *MTHFR* genotype (OR and 95 % CI) Table 3 long description.

Premenstrual Symptom	Unadjusted		Unadjusted	Adjusted		Adjusted	Unadjusted		Unadjusted	Adjusted		Adjusted	Unadjusted		Unadjusted	Adjusted		Adjusted
	OR	95 % CI	*P*	OR	95 % CI^*^	P^*^	OR	95 % CI	*P*	OR	95 % CI^*^	P^*^	OR	95 % CI	*P*	OR	95 % CI^*^	P^*^
	CC						CT						TT					
Acne/skin blemish	0·76	0·49, 1·19	0·23	0·8	0·49, 1·3	0·37	0·8	0·5, 1·28	0·35	0·82	0·49, 1·37	0·45	1·03	0·4, 2·65	0·97	0·99	0·37, 2·61	0·98
Desire to be alone	0·79	0·49, 1·26	0·32	0·74	0·44, 1·22	0·23	1·08	0·67, 1·74	0·74	1·04	0·62, 1·75	0·88	1·22	0·47, 3·18	0·68	1·37	0·51, 3·67	0·53
Anxiety/tension/nervousness	1·23	0·77, 1·95	0·38	1·4	0·85, 2·31	0·18	0·88	0·55, 1·42	0·60	1·09	0·65, 1·83	0·75	1·09	0·43, 2·76	0·86	1·37	0·52, 3·58	0·52
Increased appetite/food changes	0·92	0·58, 1·45	0·71	0·88	0·54, 1·44	0·61	0·9	0·56, 1·45	0·66	0·85	0·5, 1·43	0·53	0·93	0·35, 2·44	0·87	0·92	0·34, 2·51	0·88
Bloating/swelling/breast tenderness	0·9	0·56, 1·44	0·65	0·94	0·56, 1·57	0·81	0·94	0·53, 1·65	0·82	0·88	0·48, 1·63	0·69	0·73	0·27, 1·91	0·52	0·83	0·31, 2·27	0·72
Clumsiness	0·98	0·52, 1·84	0·95	1·18	0·6, 2·34	0·63	0·88	0·48, 1·6	0·67	1·18	0·61, 2·29	0·62	1·06	0·25, 4·59	0·94	1·38	0·31, 6·24	0·67
Confusion/difficulty concentrating/forgetfulness	0·72	0·4, 1·27	0·26	0·73	0·4, 1·36	0·33	1·2	0·71, 2·04	0·49	1·49	0·83, 2·66	0·18	1·41	0·51, 3·93	0·51	1·55	0·53, 4·52	0·42
Cramps	0·65	0·37, 1·13	0·13	0·75	0·41, 1·36	0·34	1·35	0·79, 2·33	0·27	1·39	0·77, 2·52	0·27	0·71	0·25, 1·98	0·51	0·78	0·27, 2·25	0·64
Depression	0·68	0·41, 1·13	0·14	0·72	0·41, 1·25	0·24	1·13	0·68, 1·87	0·64	1·24	0·71, 2·15	0·45	0·97	0·39, 2·46	0·96	1·26	0·48, 3·28	0·64
Fatigue	1·18	0·75, 1·84	0·47	1·06	0·65, 1·71	0·82	1·17	0·74, 1·87	0·50	1·01	0·61, 1·68	0·96	0·81	0·33, 2	0·65	0·91	0·36, 2·31	0·85
Headaches	0·78	0·48, 1·28	0·33	0·75	0·44, 1·28	0·29	0·82	0·47, 1·42	0·48	0·87	0·48, 1·59	0·65	0·52	0·18, 1·53	0·24	0·65	0·22, 1·97	0·45
Insomnia	0·93	0·46, 1·9	0·85	1·06	0·49, 2·29	0·88	1·44	0·7, 2·96	0·32	1·7	0·77, 3·76	0·19	0·49	0·11, 2·1	0·33	0·5	0·11, 2·27	0·37
Mood swings/crying easily/irritability/angry outbursts	1·06	0·65, 1·72	0·81	1·18	0·7, 1·99	0·54	0·86	0·51, 1·46	0·58	0·99	0·56, 1·75	0·97	0·93	0·34, 2·59	0·89	1·01	0·36, 2·9	0·98
Nausea	1·07	0·58, 1·97	0·82	1·15	0·59, 2·26	0·68	1·03	0·51, 2·09	0·93	1·08	0·5, 2·33	0·85	0·65	0·2, 2·03	0·45	0·96	0·29, 3·2	0·95
Sexual desire/activity change	0·98	0·63, 1·52	0·91	1·09	0·67, 1·76	0·74	0·58	0·36, 0·93	0·02	0·57	0·34, 0·96	0·03	0·64	0·26, 1·6	0·34	0·88	0·34, 2·27	0·78

* *P* value adjusted for age, log-transformed BMI, ethnicity, physical activity level, energy intake and vitamin B_12_ intake.

Excluded: smokers, hormonal imbalance diagnosis, HC users, those on antidepressants or anxiolytics, implausible energy intake (< 500 or > 3500 kcal/d) and individuals with missing information. Ref = ‘no’ premenstrual symptoms (removed for space).

**Table 4.** Interaction between *MTHFR* and folate intake premenstrual symptoms Table 4 long description.

Premenstrual symptom	*MTHFR* and folate intake interaction^*^*
Acne/skin blemish	0·92
Desire to be alone	0·42
Anxiety/tension/nervousness	0·75
Increased appetite/food cravings	0·98
Bloating/swelling/breast tenderness	0·97
Clumsiness	0·98
Confusion/difficulty concentrating/forgetfulness	0·18
Cramps	0·28
Depression	0·29
Fatigue	0·96
Headaches	0·87
Insomnia	0·89
Mood swings/ crying easily/irritability/angry outbursts	0·31
Nausea	0·96
Sexual desire/activity change	0·16

* *P*-values for the interaction between *MTHFR* and folate intake were adjusted for age, ethnicity, log-transformed BMI, physical activity levels, energy intake and vitamin B_12_ intake.

Table 5 shows the association of *MTHFR* genotype in those with folate intake below the median (< 647 mcg/d) and premenstrual symptoms. In these analyses, those with the CT genotype had increased odds of reporting premenstrual confusion/difficulty concentrating/forgetfulness (OR: 2·19; 95 % CI: 1·16, 3·80), compared with those with the CC genotype. Those with the CT genotype had higher odds of experiencing premenstrual depression, compared with those with the CC genotype (OR: 1·67; 95 % CI: 1·00, 2·82) in the univariate analyses – this finding was no longer significant when adjusted for covariates. Those with the TT genotype were at a higher risk of experiencing premenstrual depression (OR: 2·41; 95 % CI: 1·08, 5·38), after adjusting for covariates compared with those with the CC genotype. There were no further associations observed between other premenstrual symptoms and *MTHFR* genotype in women with lower folate intake.

**Table 5.** Associations between *MTHFR* genotype and premenstrual symptoms in those with lower folate intake (< 647 mcg/d) (OR and 95 % CI) Table 5 long description.

Premenstrual symptom	*MTHFR* Genotype	Unadjusted		Unadjusted	Adjusted		Adjusted
		OR	95 % CI	*P* value	OR	95 % CI^*^	*P* value^*^
Acne/skin blemish	CC						
	CT	1·23	0·77, 1·94	0·38	1·02	0·63, 1·65	0·95
	TT	1·62	0·77, 3·40	0·20	1·30	0·60, 2·85	0·50
Desire to be alone	CC						
	CT	1·47	0·91, 2·37	0·11	1·50	0·96, 2·64	0·07
	TT	1·19	0·56, 2·53	0·65	1·50	0·68, 3·30	0·31
Anxiety/tension/nervousness	CC						
	CT	0·97	0·60, 1·55	0·88	0·90	0·55, 1·48	0·68
	TT	0·99	0·47, 2·08	0·98	0·95	0·44, 2·07	0·91
Increased appetite/food cravings	CC						
	CT	0·99	0·62, 1·59	0·97	0·97	0·59, 1·59	0·90
	TT	1·35	0·63, 2·79	0·44	1·52	0·69, 3·38	0·30
Bloating/swelling/breast tenderness	CC						
	CT	1·77	1·05, 2·98	0·03	1·75	1·02, 3·00	0·04
	TT	0·91	0·43, 1·94	0·81	0·95	0·44, 2·09	0·91
Clumsiness	CC						
	CT	1·29	0·69, 2·42	0·49	1·30	0·67, 2·50	0·44
	TT	0·74	0·24, 2·29	0·60	0·68	0·21, 2·22	0·52
Confusion/difficulty concentrating/forgetfulness	CC						
	CT	2·23	1·19, 3·68	0·009	2·19	1·16, 3·80	0·01
	TT	2·22	0·98, 5·07	0·06	2·02	0·84, 4·88	0·11
Cramps	CC						
	CT	1·12	0·65, 1·93	0·67	1·19	0·67, 2·10	0·55
	TT	0·74	0·34, 1·64	0·46	0·76	0·33, 1·77	0·52
Depression	CC						
	CT	1·67	1·00, 2·82	0·05	1·66	0·96, 2·87	0·06
	TT	2·22	1·04, 4·78	0·03	2·41	1·08, 5·38	0·03
Fatigue	CC						
	CT	0·97	0·61, 1·55	0·91	1·14	0·70, 1·86	0·60
	TT	0·68	0·33, 1·39	0·28	0·85	0·40, 1·80	0·67
Headaches	CC						
	CT	0·78	0·48, 1·34	0·36	0·97	0·55, 1·72	0·92
	TT	0·65	0·27, 1·60	0·35	0·97	0·38, 2·48	0·94
Insomnia	CC						
	CT	1·38	0·68, 2·78	0·36	1·59	0·75, 3·38	0·23
	TT	0·76	0·21, 2·75	0·67	0·85	0·22, 2·24	0·81
Mood swings/crying easily/irritability/angry outbursts	CC						
	CT	1·02	0·62, 1·70	0·93	0·98	0·59, 1·68	0·96
	TT	1·06	0·47, 2·37	0·88	0·91	0·39, 2·10	0·82
Nausea	CC						
	CT	0·75	0·38, 1·45	0·38	0·71	0·36, 1·42	0·33
	TT	1·02	0·38, 2·70	0·97	1·14	0·41, 3·17	0·80
Sexual desire/activity change	CC						
	CT	0·70	0·44, 1·10	0·12	0·63	0·39, 1·03	0·06
	TT	0·63	0·30, 1·32	0·22	0·61	0·28, 1·33	0·22

* *P* value adjusted for age, log-transformed BMI, ethnicity, physical activity level, energy intake and vitamin B_12_ intake.

Excluded: individuals with folate levels ≥ 647 mcg/d, smokers, those with a hormonal imbalance diagnosis, HC users, those on antidepressants or anxiolytics, those with an implausible energy intake (< 500 or > 3500 kcal/d) and individuals with missing information.

Similar genetic association analyses were carried out between *MTHFR* genotype and individual premenstrual symptoms, but in those above the median folate intake (≥ 647 mcg/d) and are presented in Table 6. In this analysis, we found that women with higher folate intake, who have the CT genotype of *MTHFR*, had increased odds of reporting premenstrual bloating/swelling/breast tenderness, compared with those with the CC genotype after adjusting for covariates (OR: 1·93; 95 % CI: 1·09, 3·21). Women who consumed higher folate, and who had the CT genotype, had lower odds of experiencing premenstrual cramps, compared with those with CC genotype (OR: 0·54; 95 % CI: 0·31, 0·94). However, this association was no longer observed after adjusting for covariates.

**Table 6.** Associations between *MTHFR* genotype and premenstrual symptoms in those with higher folate intake (≥ 647 mcg/d) (OR and 95 % CI) Table 6 long description.

Premenstrual symptom	*MTHFR* genotype	Unadjusted		Unadjusted	Adjusted		Adjusted
		OR	95 % CI	*P* value	OR	95 % CI^*^	*P* value^*^
Acne/skin blemish	CC						
	CT	1·17	0·73, 1·86	0·52	1·03	0·63, 1·70	0·90
	TT	1·18	0·57, 2·46	0·65	1·15	0·53, 2·48	0·72
Desire to be alone	CC						
	CT	1·07	0·67, 1·71	0·77	1·16	0·70, 1·92	0·57
	TT	0·77	0·36, 1·64	0·50	0·85	0·39, 1·87	0·69
Anxiety/tension/nervousness	CC						
	CT	1·35	0·84, 2·16	0·21	1·24	0·75, 2·05	0·41
	TT	1·12	0·55, 2·34	0·76	1·11	0·51, 2·39	0·79
Increased appetite/food cravings	CC						
	CT	1·01	0·64, 1·62	0·95	1·02	0·61, 1·69	0·95
	TT	1·33	0·63, 2·85	0·45	1·47	0·66, 3·25	0·34
Bloating/swelling/breast tenderness	CC						
	CT	1·69	1·00, 2·86	0·05	1·93	1·09, 3·21	0·02
	TT	1·13	0·52, 2·46	0·76	1·12	0·50, 2·54	0·77
Clumsiness	CC						
	CT	1·44	0·78, 2·65	0·24	1·30	0·66, 2·51	0·44
	TT	0·68	0·22, 2·11	0·50	0·62	0·19, 2·01	0·43
Confusion/difficulty concentrating/forgetfulness	CC						
	CT	1·25	0·73, 2·14	0·42	1·28	0·71, 2·29	0·40
	TT	1·13	0·49, 2·64	0·77	1·29	0·53, 3·12	0·57
Cramps	CC						
	CT	0·54	0·31, 0·94	0·02	0·66	0·36, 1·20	0·17
	TT	0·68	0·29, 1·61	0·38	0·70	0·29, 1·73	0·44
Depression	CC						
	CT	1·00	0·61, 1·66	0·98	1·08	0·63, 1·86	0·77
	TT	1·55	0·74, 3·23	0·24	1·46	0·67, 3·18	0·34
Fatigue	CC						
	CT	0·98	0·63, 1·54	0·92	1·15	0·70, 1·88	0·57
	TT	0·98	0·48, 1·20	0·96	0·92	0·44, 1·94	0·82
Headaches	CC						
	CT	0·75	0·45, 1·24	0·26	0·77	0·45, 1·33	0·35
	TT	0·97	0·45, 2·08	0·94	1·01	0·45, 2·25	0·98
Insomnia	CC						
	CT	0·90	0·43, 1·87	0·77	0·95	0·42, 2·14	0·90
	TT	1·45	0·53, 3·98	0·47	1·86	0·63, 5·50	0·26
Mood swings/crying easily/irritability/angry outbursts	CC						
	CT	1·26	0·76, 2·10	0·37	1·12	0·65, 1·96	0·68
	TT	1·21	0·54, 2·70	0·64	1·16	0·50, 2·68	0·73
Nausea	CC						
	CT	0·78	0·40, 1·51	0·45	0·84	0·41, 1·73	0·64
	TT	1·70	0·71, 4·05	0·23	1·54	0·60, 3·95	0·36
Sexual desire/activity change	CC						
	CT	1·18	0·75, 1·85	0·49	1·30	0·80, 2·14	0·30
	TT	0·96	0·47, 1·95	0·92	0·87	0·41, 1·84	0·72

* *P* value adjusted for age, log-transformed BMI, ethnicity, physical activity level, energy intake and vitamin B_12_ intake.

Excluded: individuals with folate levels < 647 mcg/d, smokers, those with a hormonal imbalance diagnosis, HC users, those on antidepressants or anxiolytics, those with an implausible energy intake (<500 or >3500 kcal/d) and individuals with missing information.

## Discussion

The purpose of this study was to examine the association between folate, using dietary measures and genetic variation in *MTHFR*, and premenstrual symptoms. We observed that in women with lower folate intake, carriers of the Tallele had increased odds of reporting premenstrual depression, compared with those with the CC genotype. To our knowledge, this is the first study to investigate the role of *MTHFR* in premenstrual symptoms.

Variations in the *MTHFR* gene determine the way individuals use dietary folate. The most commonly researched genetic variant affecting folate metabolism, a variant in the *MTHFR* gene (rs1801133) involving a cytosine to thymidine (C → T) transition at nucleotide 677, is associated with reduced enzyme activity, DNA hypomethylation, developmental anomalies and elevated plasma homocysteine levels, which have been shown to increase the risk of cardiovascular disease^(35)^. Individuals with the TT genotype for the C677T polymorphism have increased plasma homocysteine and lower serum folate concentrations compared with those who have the CC genotype and heterozygotes appear to have an intermediate phenotype^(11,13,15,35)^. Many polymorphisms in genes involved in folate and homocysteine metabolism have been examined for their effects on folate and homocysteine levels, but the most consistent, and the largest effect is observed with the *MTHFR C677T* variant^(37,38)^.

Folate is involved in neurotransmitter synthesis directly through its biologically active form, L-methyl-folate^(39)^. This active form of folate is also involved in converting homocysteine to methionine, which then converts to S-adenosyl-methionine, which is an important methyl donor in the synthesis of norepinephrine, dopamine and serotonin – all of which are neurotransmitters that play a role in depression^(40,41)^. Our research is consistent with previous findings on folate and depression^(18,39)^ and further extends this relationship to our understanding of premenstrual depression and folate. A meta-analysis of randomised control trials examined the efficacy of adjunctive folate for major depressive disorders and suggested that folate was superior to placebo in treating depressive disorders and other major mental health disorders such as bipolar manic episodes^(20)^. Another meta-analysis examined the relationship between *MTHFR* (C677T) genotype and depression diagnosis and reported that those homozygous for the Tallele of the *MTHFR* gene compared with CC individuals had higher odds of being diagnosed with depression^(19)^. These findings are in line with previous studies showing those with the TT genotype of *MTHFR* genotype have a higher chance of being diagnosed with depression. Taking into consideration *MTHFR* genotype and folate status may be useful in clinical settings when assessing depressive disorders.

Premenstrual depression is a major symptom of PMDD, a diagnostic category of depressive disorders classified in the 5th Diagnostic and Statistical Manual of Mental Disorders^(4)^. PMDD is also coded as a gynaecological diagnosis in the WHO’s International Classification of Diseases^(42)^. The estimated prevalence of PMDD in the USA is around 8 %^(43)^, and in southern Brazil, this estimate goes up to 17·6 %^(44)^. These rates continue to rise, worldwide. Outside of the context of menstrual disorders, women who suffer from premenstrual symptoms, are more likely to suffer from postpartum depression as well^(45–47)^. Interestingly, there seems to be inconsistent evidence for folate as a therapeutic factor for postpartum depression. Some epidemiological studies have found no relationships between folate and postpartum depression^(48)^, while systematic reviews have found evidence for folate as a therapeutic measure for perinatal depression^(49)^. These inconsistencies may be due to genetic variation in folate metabolism and due to the increased intake of folic acid during the perinatal period and fortification of folic acid in some countries. Despite the mandatory folate fortification of wheat flour and cornmeal in North America, to our knowledge, there is no research directly assessing populations with and without fortification and mental health outcomes. Given the high prevalence of depression as a symptom within menstrual disorders, and in the context of women’s health, it is vital to find minimally invasive lifestyle therapies, such as dietary changes, to decrease the risks of depression in these populations.

The present study has several strengths as well as some limitations. We used different measurements of folate including dietary intake and genetic variation in folate metabolism. Using genetic measures of folate metabolism provides an unbiased approach to evaluating the role of folate in premenstrual symptoms. However, the TNH study did not measure plasma or red blood cell folate concentrations, which could have provided a more comprehensive view of folate status. Though participants of the TNH study belonged to one of the three major ethnic groups in Canada, we are left with minimal information on those from Indigenous communities and black individuals. The GHLQ from the TNH study also gathered a comprehensive set of information on each participant’s lifestyle habits, which allowed us to adjust for multiple factors that are recognised to affect both premenstrual symptoms and folate status, minimising the likelihood of residual confounding. Moreover, the association between a polymorphism and nutrient levels is not subject to the limitations of confounding and reverse causation inherent in examining associations between lifestyle factors and health outcomes^(50)^. Thus, associations found between *MTHFR* C677T and premenstrual depression add to the evidence implicating folate and its metabolites in this condition.

In conclusion, the current study’s findings indicate that *MTHFR* genotype may be associated with premenstrual depression specifically in those who have lower intakes of dietary folate. Future research should continue to assess the associations between folate and other B-vitamins and premenstrual depression in intervention settings in women with premenstrual symptoms, PMS and PMDD and account for *MTHFR* genotype. Furthermore, more research utilising precision nutrition in this field is necessary to provide personalised dietary recommendations.

## Figure 1 Long description

The flowchart begins with 1649 participants from the Toronto Nutrigenomics and Health Study. Exclusions are made for males (532), those using hormonal contraceptives (304), smokers (52), individuals on anti-depressants or anxiolytics (24), those with reported diagnoses of endometriosis, amenorrhea, or polycystic ovarian syndrome (20), participants with missing data (3), and those with implausible energy intake (36). After these exclusions, the total sample size is reduced to 678.

Navigate back to Figure 1.

## Table 1 Long description

The table presents participant characteristics stratified by MTHFR genotype, with columns for CC, CT, and TT genotypes. It includes data on the number and percentage of participants, mean age, ethnicity distribution, body mass index (BMI), physical activity level, and daily intake of energy, carbohydrates, total fat, protein, thiamin, riboflavin, niacin, pantothenic acid, vitamin B6, folate, and vitamin B12. Notable trends include higher energy, protein, and total fat intake among participants with the TT genotype compared to those with the CT and CC genotypes.

Navigate back to Table 1.

## Table 2 Long description

The table presents data on the associations between folate intake and various premenstrual symptoms. It includes columns for folate intake levels, percentages, unadjusted and adjusted odds ratios, and confidence intervals. The table has 17 rows and 7 columns, covering symptoms such as acne, desire to be alone, anxiety, increased appetite, bloating, clumsiness, confusion, cramps, depression, fatigue, headaches, insomnia, mood swings, nausea, and changes in sexual desire. Each row provides the number and percentage of participants experiencing each symptom, stratified by folate intake levels below and above the median of 647 micrograms per day. The table also includes unadjusted and adjusted odds ratios with 95 percent confidence intervals, indicating no significant associations between folate intake and any premenstrual symptoms in both univariate and multivariate models.

Navigate back to Table 2.

## Table 3 Long description

The table presents the associations between total folate intake and various premenstrual symptoms, stratified by MTHFR genotype. It includes unadjusted and adjusted odds ratios (OR) with 95 percent confidence intervals (CI) and P values for three different genotypes: CC, CT, and TT. The table has 16 rows, each representing a different premenstrual symptom, and 12 columns displaying unadjusted and adjusted OR, CI, and P values for each genotype. Notable symptoms include acne, desire to be alone, anxiety, increased appetite, bloating, clumsiness, confusion, cramps, depression, fatigue, headaches, insomnia, mood swings, nausea, and changes in sexual desire. The data indicates no significant associations between folate intake and any premenstrual symptoms when stratified by MTHFR genotype. Additionally, MTHFR did not modify the association between folate intake and premenstrual symptoms after adjusting for covariates.

Navigate back to Table 3.

## Table 4 Long description

The table presents data on the interaction between MTHFR and folate intake on various premenstrual symptoms. It consists of 15 rows and 2 columns. The first column lists premenstrual symptoms such as acne, desire to be alone, anxiety, increased appetite, bloating, clumsiness, confusion, cramps, depression, fatigue, headaches, insomnia, mood swings, nausea, and sexual desire changes. The second column shows the interaction values between MTHFR and folate intake for each symptom, ranging from 0.16 to 0.98. The table provides a detailed comparison of how different symptoms are influenced by the interaction between MTHFR and folate intake.

Navigate back to Table 4.

## Table 5 Long description

The table presents the association of MTHFR genotype with various premenstrual symptoms in individuals with folate intake below the median of 647 micrograms per day. The table includes unadjusted and adjusted odds ratios (OR), 95 percentage confidence intervals (CI), and P values for different genotypes (CC, CT, TT) across multiple premenstrual symptoms. Key findings include increased odds of premenstrual confusion, difficulty concentrating, or forgetfulness for those with the CT genotype compared to the CC genotype. Additionally, those with the TT genotype show a higher risk of premenstrual depression after adjusting for covariates. The table highlights specific associations and the impact of folate intake on premenstrual symptoms.

Navigate back to Table 5.

## Table 6 Long description

A table with thirteen rows and five columns presents data on the associations between MTHFR genotype and various premenstrual symptoms in women with higher folate intake. The columns are labeled MTHFR genotype, Unadjusted OR, Unadjusted 95 percentage CI, Unadjusted P value, Adjusted OR, Adjusted 95 percentage CI, and Adjusted P value. The rows list different premenstrual symptoms such as acne/skin blemish, desire to be alone, anxiety/tension/nervousness, increased appetite/food cravings, bloating/swelling/breast tenderness, clumsiness, confusion/difficulty concentrating/forgetfulness, cramps, depression, fatigue, headaches, insomnia, mood swings/crying easily/irritability/angry outbursts, nausea, and sexual desire/activity change. The table provides odds ratios, confidence intervals, and p-values for each symptom across different genotypes (CC, CT, TT) under unadjusted and adjusted conditions. Notable trends include increased odds of bloating/swelling/breast tenderness for the CT genotype compared to the CC genotype after adjustment, and lower odds of premenstrual cramps for the CT genotype compared to the CC genotype before adjustment.

Navigate back to Table 6.
